# Research progress and challenges of the PD-1/PD-L1 axis in gliomas

**DOI:** 10.1186/s13578-024-01305-6

**Published:** 2024-09-27

**Authors:** Dong Jiacheng, Cui Jiayue, Guo Ying, Wang Shaohua, Liu Wenhui, Hong Xinyu

**Affiliations:** 1 Department of Neurosurgery, Jilin Provincial Hospital, The First Hospital of Jilin University, 1 Xinmin Street, Changchun, Jilin, 130021 China; 2 Department of Histology and Embryology, The School of Basic Medicine, Jilin University, 126 Xinmin Street, Changchun, Jilin, 130021 China; 3 Department of Infectious Diseases, Infectious Diseases and Pathogen Biology Center, The First Hospital of Jilin University, Changchun, Jilin, 130021 China

**Keywords:** Glioma, PD-1, PD-L1, Microenvironment, Immune cells, Combination therapy

## Abstract

The emergence of programmed death-1 (PD-1) and programmed death ligand 1 (PD-L1) immunosuppressants provides new therapeutic directions for various advanced malignant cancers. At present, PD-1/PD-L1 immunosuppressants have made significant progress in clinical trials of some gliomas, but PD-1/PD-L1 inhibitors have not yet shown convincing clinical efficacy in gliomas. This article summarizes the research progress of the PD-1 /PD-L1 pathway in gliomas through the following three aspects. It mainly includes the complex expression levels and regulatory mechanisms of PD-1/PD-L1 in the glioma microenvironment, the immune infiltration in glioma immunosuppressive microenvironment, and research progress on the application of PD-1/PD-L1 immunosuppressants in clinical treatment trials for gliomas. This will help to understand the current treatment progress and future research directions better.

## Introduction

Glioma is one of the most common intracranial tumors worldwide, characterized by high invasiveness [1]. The current standard treatment regimen for glioma is a multimodal approach that combines maximum surgical resection, radiotherapy, and chemotherapy [2, 3]. However, due to its high invasiveness and inherent therapeutic resistance, the recurrence rate is extremely high [2, 4]. The treatment options for recurrent gliomas are limited and the prognosis is poor [3]. Therefore, it is necessary to find new therapeutic approaches for the diagnosis and treatment of recurrent gliomas.

A prominent feature of glioma is that local and systemic immunosuppression occurs within the tumor microenvironment (TME), thereby eliminating anti-tumor immune defense [5]. This feature makes immunotherapy a focus of attention. Cancer immunotherapy recognizes, targets, and destroys cancer cells by restoring or inducing the ability of immune system effector cells [6]. Immune checkpoint inhibitors, as an emerging immunotherapy, have sparked a wave in many tumor treatment studies and have made significant clinical progress. Among them, PD-1/PD-L1 axis has garnered extensive research interest.

At present, PD-1/PD-L1 immune checkpoint inhibitors have been considered for the treatment of tumors due to their significant role in remodeling the tumor microenvironment and releasing PD-1/PD-L1 binding inhibitory effects in various histological tumors. PD-1/PD-L1 has been shown to be specifically expressed on different cell types in TME [7], therefore investigating the role of PD-1/PD-L1 is helpful for the diagnosis and treatment of glioma patients.

## Expression, distribution and regulation of PD-L1 in glioma microenvironment

### Expression and distribution of PD-L1 in glioma microenvironment

PD-1 is an important immunosuppressive receptor, mainly located on the surface of activated CD3^+^/CD8^+^T cells, macrophages, B lymphocytes, dendritic cells (DC), monocytes, tumor specific activated T cells, myeloid cells and natural killer cells (NK) [8, 9]. The expression of PD-1 mainly involves innate and adaptive immune components. The PD-1/PD-L1 pathway participates in inhibitory TME by inhibiting T cell activation and proliferation, inhibiting immune cell recruitment, and regulating the expression of other immune checkpoints. PD-L1 is a ligand of PD-1, mainly expressed in tumor cells and antigen presenting cells (APCs) and others in tumor tissue [10]. Relevant study havs shown that the level of PD-L1 in cancer cells is considered to be the main predictor of PD-1/PD-L1 antibody response [11]. The expression of PD-1/PD-L1 in gliomas provides important predictive value for immunosuppressive treatment of gliomas. PD-L1 expression is up-regulated in a variety of tumors, and high expression levels of PD-L1 often indicate better clinical efficacy of PD-1/PD-L1 checkpoint blocking [12–15]. Therefore, detection of PD-L1 expression can help clinicians predict the possible response of glioma patients to immunosuppressive therapy, and provide a basis for individualized treatment decisions, which has important clinical therapeutic significance.

To date, the available evidence suggests that the expression level of PD-L1 in gliomas is directly proportional to the glioma grade [16, 17]. Moreover, the expression of PD-L1 in the edge of glioma tumor cells is significantly higher than that in the tumor core [18]. High levels of PD-L1 expression contribute to immune escape of glioma cells. The high level of peripheral expression of gliomas further indicates that the up-regulation of PD-L1 at tumor margins can form a barrier between tumor cells and immune cells. This phenomenon helps tumors evade immune surveillance when invading adjacent brain tissue. Meanwhile, other study has found that the expression of PD-1/PD-L1 in patients with recurrent glioma is significantly higher than that in patients with newly diagnosed glioma, and the increase in PD-L1 level is closely related to receiving adjuvant therapy such as radiotherapy and chemotherapy [19].

### Regulation of PD-L1 expression in glioma microenvironment

**Fig. 1:**
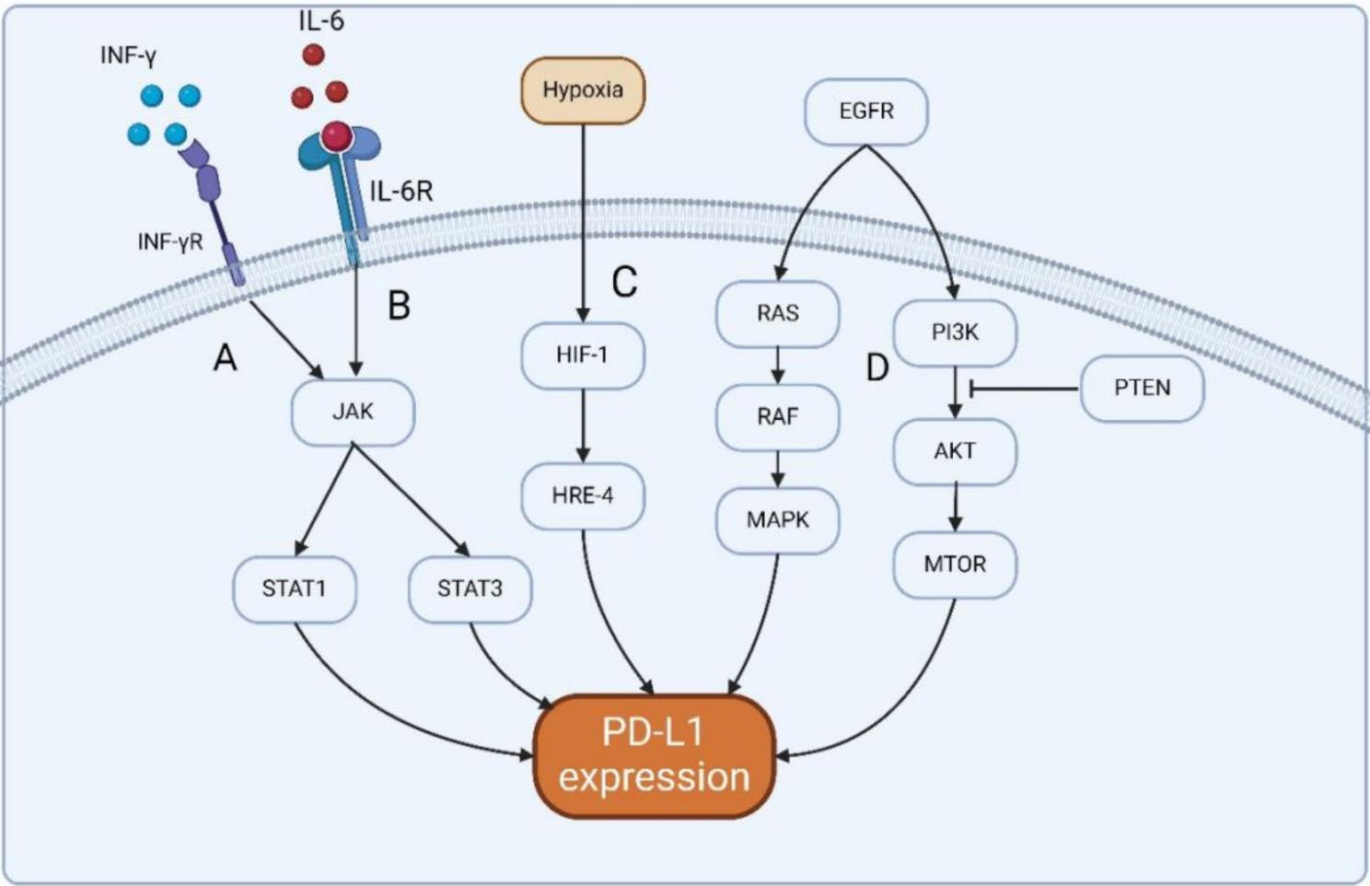
Regulation of PD-L1 expression in glioma microenvironment. (**A**) INF-γ can bind to the INF-γ receptor on the cell surface, and the activated receptor activates the JAK protein kinase. Activated JAK protein kinase phosphorylates STAT (signal transduction and transcriptional activator) proteins, mainly inducing STAT1 and STAT3. The phosphorylated STAT1 protein forms a STAT1 dimer that binds to the promoter region of the PDL1 gene. In this way, STAT1 dimerization promotes the transcription of PD-L1 gene and expresses PD-L1 protein.(**B**) IL-6 can also induce PD-L1 expression through STAT3 phosphorylation via the JAK /STAT3 pathway.(**C**) HIF-1 can regulate PD-L1 expression by directly binding to hypoxia responsive element-4 (HRE-4) in the proximal promoter of PD-L1.(**D**) EGFR via TNF-αActivate or combine with EGF to induce Ras /RAF /MAPK and PI3K /Akt /mTOR signaling pathways to promote PD-L1 expression. The PTEN gene can regulate the expression of PD-L1 in gliomas by negatively regulating the activation of Akt in the PI3K /Akt /mTOR signaling pathway

In the glioma microenvironment, PD-L1 is regulated by many cytokines. For example, epidermal growth factor receptor(EGFR) is activated by tumor growth factor-α(TNF-α)or epidermal growth factor (EGF), inducing Ras/RAF/MAPK and PI3K/Akt/mTOR signaling pathways to promote PD-L1 expression [20]. PTEN gene negatively regulates the activation of Akt in the PI3K/Akt/mTOR signaling pathway, which plays an important role in regulating the expression of PD-L1 in glioma (Fig. 1). Homozygous deletion or mutation of PTEN is found in glioma, and it is positively correlated with PD-L1 expression [20]. In addition, the immunoinducible cytokines interferon-γ (IFN-γ) and interleukin-6 (IL-6) can also induce the expression of PD-L1 in the glioma microenvironment. IFN-γ is the most effective inducer of PD-L1 expression, mainly derived from T cells or NK cells [21]. INF-γ Can interact with the INF-γ Receptor on the cell surface activates the expression of PD-L1 (Fig. 1). The PD-L1 protein will locate to the cell membrane and bind to the PD-L1 receptor. IL-6 can also induce PD-L1 expression by phosphorylation of STAT through the JAK/STAT pathway (Fig. 1) [22]. In glioma TME, the cytokines IFN-γ and IL-6 can act on immune cells such as glioma cells and phagocytes to increase the expression of PD-L1, and finally bind to PD-1 on TIL to inhibit the activation and proliferation of T cells, helping to maintain immune tolerance and prevent the occurrence of autoimmune reactions.

In addition, hypoxia can also induce cells to express more PD-L1, because hypoxia-inducible factor-1 (HIF-1) plays a key role in regulating the cell response to hypoxia. HIF-1 can regulate PD-L1 expression by directly binding to hypoxia responsive element-4 (HRE-4) in the proximal promoter of PD-L1 [23]. Hypoxia upregulates PD-L1 by increasing HIF-1α in glioma cells [24].

In general, PD-L1 expression in the glioma microenvironment is primarily governed by various cytokines, forming a intricate regulatory network. Upregulated PD-L1 can create an immunosuppressive environment by interacting with PD-1 on immune cells, impacting glioma treatment and patient prognosis.

## PD-L1 regulates immune component infiltration in glioma TME

TME is an important factor affecting the occurrence, immunosuppression, and progress of glioma. There are tumor cells and a large number of non-tumor cells in TME. Non-tumor cells mainly include tumor-infiltrating lymphocytes (TILs) and glioma-associated macrophages (GAMs). Non-tumor cells play a crucial role in the proliferation and infiltration of glioma cells. The PD-1/PD-L1 axis can alter the state of non-tumor cells in gliomas, forming a glioma immunosuppressive microenvironment. Therefore, studying the mechanisms by which various immune components in glioma form an immunosuppressive microenvironment plays a crucial role in the clinical treatment of glioma patients. The immune cells associated with the PD-1/PD- L1 axis that regulates immune infiltration are as follows.

### Glioma associated macrophages (GAMs)

**Fig. 2:**
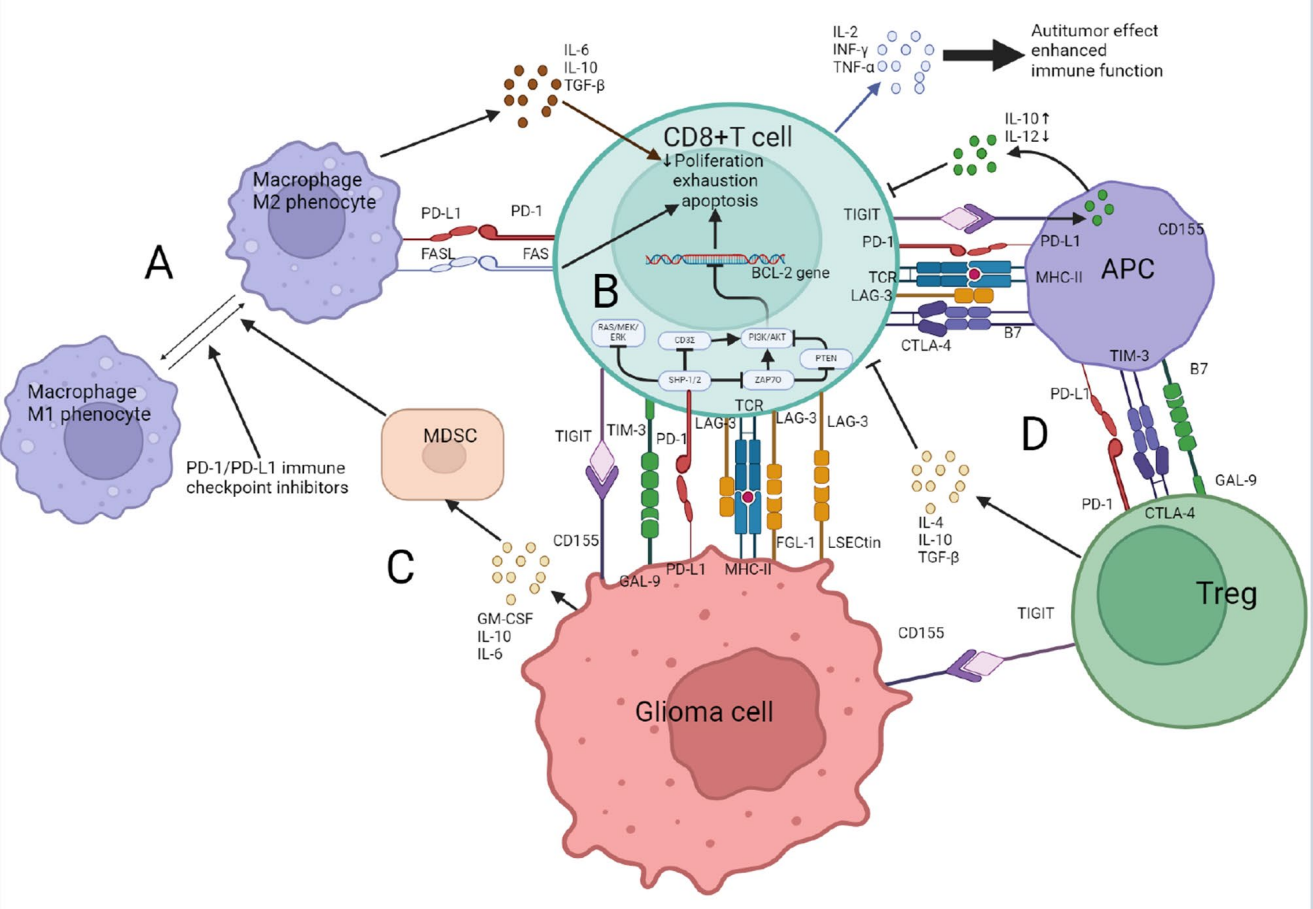
PD-1/PD-L1signaling pathway mediated immune escape mechanism in glioma immunosuppressive microenvironment. **(A)** Glioma-associated macrophages (GAMs), primarily of the M2 subtype, are influenced by cytokines such as IL-6, IL-10, and TGF-β, which modulate the immune response, induce lymphocyte apoptosis, and help glioma cells evade the immune system. GAMs can upregulate FasL and PD-L1 during antigen presentation, interacting with CD8+ T cells to trigger programmed cell death. **(B)** The PD-1/PD-L1 signaling pathway in cytotoxic T lymphocytes (CTLs) is initiated following T cell receptor (TCR) activation by tumor-specific antigens. PD-1 binds to PD-L1, leading to the recruitment of Src Homology 2 domain-containing protein tyrosine phosphatase (SHP)-1 and SHP-2, which dephosphorylate TCR-activated signaling molecules such as ZAP70 and CD-3ζ. This results in the inhibition of the phosphoinositide 3-kinase (PI3K)/protein kinase B (Akt) pathway, leading to downstream effects that include the down-regulation of Bcl-2 family genes, promotion of CTL apoptosis, and inhibition of cytokine secretion (IFN-γ, TNF-α, and IL-2). Furthermore, the PD-1/PD-L1 interaction inhibits tyrosine phosphatase activity and reduces RAS protein phosphorylation, thus impeding the RAS/MEK/ERK pathway and inhibiting T cell proliferation and cell cycle progression, ultimately causing T cell apoptosis. **(C)** Glioma cells secrete cytokines like GM-CSF, IL-6, and IL-10, which promote the expansion of myeloid-derived suppressor cells (MDSCs). MDSCs can convert macrophages from the pro-inflammatory M1 to the tumor-supportive M2 subtype, contributing to CTL apoptosis. **(D)** Regulatory T cells (Tregs) release inhibitory cytokines such as transforming growth factors- β (TGF- β) And IL-10, suppressing T lymphocyte activity. Tregs also interact with antigen-presenting cells (APCs) through CTLA-4 and PD-1, inhibiting their function and reducing the ability of APCs to present antigens, which indirectly impairs T lymphocyte function and suppresses the immune response

The most abundant immune cells in TME are GAMs, accounting for 30–50% of massive glioma cells [25]. GAMs have strong plasticity and can polarize into different phenotypes situation in different microenvironments. GAMs are generally divided into pro-inflammatory M1 subtype and anti-inflammatory M2 subtype. Current research shows that the main subtype of GAMs in gliomas is M2 subtype [26], which can mediate lymphocyte dysfunction, depletion, and apoptosis through various pathways, weaken anti-tumor immunity, and promote tumor immune escape (Fig. 2) [27]. Different GAM phenotypes affect anti-PD-1 therapy for gliomas, with early-stage M1 subtype activation aiding the response, while advanced M2 subtype transformation may cause resistance, linked to increased PD-1/PD-L1 expression [28]. In addition, PD-L1 has been found to provide reverse signaling to inhibit the anti-glioma activity of PD-L1 expressing T cells [29]. Therefore, it can be speculated that while PD-L1 expression increases during the transformation of GAMs from M1 to M2 subtype, PD-L1 can also limit M1-phenotype activation of GAMs.

### Tumor-infiltrating lymphocyte (TILs)

TILs are another important component of glioma immune infiltration, mainly include CD8^+^T cells and CD4^+^T cells. Tumor-infiltrating CD8^+^T cells are the key cells to clearing glioma cells, which can inhibit the invasive growth of glioma by secreting cytokines. Therefore, one of the key features of glioma is the depletion and impaired function of T cells. The PD-1 / PD-L1 axis induces the functional exhaustion, apoptosis of tumor-infiltrating CD8^+^T cells (cytotoxic T lymphocytes (CTL)) to exert an immunosuppressive effect. The high expression of PD-1/PD-L1 axis can cause dysfunction of CD8^+^T cells in glioma TME. The interaction between PD-L1 on glioma and PD-1 on CD8 + T cells in glioma TME leads to dysfunction of CD8 + T cells through multiple pathways of action (Fig. 2) [30]. And the expression of PD-L1 in glioblastoma patients without anticancer therapy significantly down-regulates the intensity of glioma infiltrating CD8^+^T cells [31]. It is indicates that the key to glioma treatment is lowing the expression of PD-1 in tumor infiltrating CD8^+^T cells.

For CD4^+^T cells, the PD-1/PD-L1 axis leads to the loss of normal function and imbalance of cytokine secretion [32]. And the binding of PD-L1 and PD-1 on CD4^+^T cells induces their transformation into CD4^+^CD25^+^FoxP3^+^regulatory T cells (an effective subset of immunosuppressive effector T cells), mediating the regulation of immunosuppressive factors to weaken effector T cell activity [33]. Glioma can evade immune surveillance and eradication by upregulating PD-L1 expression, thereby utilizing and selecting the PD-1/PD-L1 axis as a shield for maladaptation, which is beneficial for tumor growth [34]. This constitutes one of the multiple mechanisms of glioma immune evasion.

### Regulatory T cells (Tregs)

It is known that induction of Tregs accumulate in the TME of glioma [35]. Treg can inhibit the proliferation and degranulation of CTL through various pathways, which helps to form a glioma immunosuppressive microenvironment (specific process can be seen in Fig. 2). Study has shown that PD-L1 may expand and maintain immunosuppressive Tregs, which are associated with decreased survival in glioma patients [36]. Thus, blocking the PD-L1/PD-1 axis has a direct effect on effector T cells and may also reduce Treg amplification and further improve T cell function, enhancing anti-tumor immune response. In TME of gliomas, the PD-1/PD-L1 axis has multiple inhibitory effects on the immune system. Therefore, the combination therapy of immune checkpoint inhibitors and methods to reactivate the immune system, such as TAM repolarization and T lymphocyte regeneration, maybe more helpful for clinical efficacy.

### Tumor-infiltrating B cells

Relevant studies have shown that B cells mainly play protumorigenic functions in glioma TME, showing immunosuppressive effects on CD8 + T cells, which is completely opposite to the anti-tumor function of peripheral B cells [37, 38]. The myeloid-derivedsuppressorcells(MDSC) play an indispensable role in the development of immunosuppressive phenotype in B cells. MDSC can transform normal B cells into Bregs, which can inhibit the immune response, and MDSC can mediate the transfer of membrane-bound PD-L1 to B cells [38, 39]. Bregs can inhibit the proliferation and activation of T cells through the combination of PD-L1 and PD-1 on T cells. Meanwhile, the interaction between Bregs and T cells is that Bregs overexpresses TGF-β and IL-10, which can also inhibit the proliferation and immune activity of T cells [38]. This phenomenon indicates that Bregs can inhibit the proliferation and activation of T cells and maintain an immunosuppressive environment in glioma TME.

### Myeloid-derived suppressor cells (MDSCs)

MDSCs are a heterogeneous population of immunosuppressive cells derived from myeloid progenitor cells and immature myeloid cells(IMCs). Under normal physiological conditions, IMCs rapidly develop into mature macrophages, granulocytes, or dendritic cells, which are involved in the immune response [40]. However, in glioma TME, chronic inflammatory disease prevents IMC from differentiating into mature bone marrow cells, leading to a continuous accumulation of MDSC in the TME [41]. There are three main types of MDSC: granulocyte or pleomorphic nuclear MDSC (G/PMN-MDSC), mononuclear MDSCs (M-MDSC), and early-stage MDSCs (eMDSC). Relevant studies have shown that in glioma patients, M-MDSC is mainly present in human blood, while PMN-MDSC is dominant in glioma TME [42, 43]. In glioma TME, glioma cells can secrete granulocyte macrophage colony-stimulating factor(GM-CSF) [44], IL-6 [44], IL-10 [45] and other cytokines to stimulate the expansion of MDSC. The effect of MDSC amplification in TME is to induce immunosuppression to promote the growth and development of glioma. Immunosuppression is mainly induced by the expression of PD-L1 on MDSC can bind to PD-1 on the surface of CTL, inhibit the initiation of anti-tumor immune response of CTL, and lead to the functional inactivation of CTL [46]. MDSC can also tilt macrophages from M1-subtype to M2-subtype by making cell-to-cell contact with macrophages [47], ultimately leading to an increase in M2-subtype leading to apoptosis of CIL (Fig. 2). MDSC in TME also converts normal B cells into Bregs, which suppress the immune response. In addition, MDSC can directly support the growth of tumors (or cancer stem cells) and promote the growth of cancer cells by affecting angiogenesis, invasion, and metastasis [48].

In summary, the mechanism of sustained growth, development and infiltration of glioma cells in TME is the mutual regulation of a variety of immune cells and immune regulatory cytokines to form an immunosuppressive microenvironment, in which the PD-1/PD-L1 signaling pathway plays an indispensable role. The immunosuppressive microenvironment contributes to the further development of glioma and affects the prognosis and treatment response of glioma patients. Therefore, the investigation of the immune escape mechanism mediated by PD-1/PD-L1 signaling pathway in glioma immune microenvironment is conducive to the optimization and further exploration of clinical treatment programs.

## PD-1/PD-L1 axis is applied in clinical treatment

In recent years, PD-1/PD-L1 has greatly improved the treatment mode of a variety of malignant tumors, such as melanoma, small cell lung cancer, urothelium carcinoma, colorectal cancer, and so on [49–51]. With the continuous understanding of glioma self-tolerance and immunosuppressive microenvironment, PD-1/PD-L1 immunosuppressive inhibitors provide a new approach for the treatment of glioma.

### Anti-PD-1/PD-L1 monotherapy

At present, PD-1/PD-L1 immunosuppressants used in clinical tumor treatment mainly include Pembrolizumab, Nivolumab, Pabolizumab, etc [52]. Table 1 shows the PD-1/PD-L1 targeted inhibitors in a large number of studies in newly diagnosed and recurrent gliomas. PD-1/PD-L1 inhibitiors has been demonstrated to restore anti-tumor T-cell activity, induce tumor cell regression, and improve survival rates, thus paving the way for clinical trials. Preclinical glioma models have shown the efficacy of PD-1/PD-L1 inhibitors in restoring immune cell activity in the glioma TME, leading to tumor regression and improved survival [53, 54]. PD-L1 inhibitors in combination with stereotactic radiosurgery significantly improved survival in mice, with a median OS of 52 days compared to 27 and 30 days of OS treated with SRS or PD-1 inhibitors alone [55]. Relevant clinical studies have shown that 24 glioma patients treated with pembrolizumab had a median PFS of 1.4 months and a median OS of 4 months. This suggests that patients with glioma may benefit from the use of PD-1/PD-1 immunotherapy [56]. Research on glioma patients has shown that Pembrolizumab exhibits anti-tumor activity, which helps to treat and improve the prognosis of glioma patients [57]. However, clinical study has shown limited efficacy with single-agent PD-1/PD-L1 therapy. A clinical trial reports suggests limited survival benefit in glioma patients receiving pembrolizumab monotherapy [58]. In summary, in view of the current status of PD-1/PD-L1 immune checkpoint treatment for glioma, it is necessary to further analyze the causes and explore new therapeutic strategies, such as combination therapy.

**Table 1:** Clinical study of PD-1/PD-L1 inhibitors in Glioma

NCT Number	Study Status	Study Results	Phases	Population	Dates	Traget	Checkpoint Inhibitor
NCT02658279	ACTIVE ; NOT RECRUITING	NO	Not Applicable	27	2016/1/22-2025/1/1	PD-L1	Pembrolizumab
NCT05188508	RECRUITING	NO	2	57	2022/1/14-2025/1/1	PD-L1	Pembrolizumab
NCT02311582	COMPLETED	NO	1\2	55	2015/8/5-2025/1/1	PD-L1	Pembrolizumab
NCT05235737	RECRUITING	NO	4	36	2022/3/1-2026/5/30	PD-L1	Pembrolizumab
NCT03899857	ACTIVE ; NOT_RECRUITING	NO	2	56	2020/10/21-2026/12/1	PD-L1	Pembrolizumab
NCT02337491	COMPLETED	YES	2	80	2015/2/9-2020/9/14	PD-L1	Pembrolizumab
NCT04118036	WITHDRAWN	NO	2	0	2021/12/1-2024/12/1	PD-L1	Pembrolizumab
NCT03557359	ACTIVE ; NOT_RECRUITING	NO	2	20	2018/6/12-2026/12/1	PD-1	Nivolumab
NCT03718767	RECRUITING	NO	2	70	2019/3/27-2026/2/27	PD-1	Nivolumab
NCT03925246	COMPLETED	NO	2	43	2019/7/30-2021/8/18	PD-1	Nivolumab
NCT02960230	COMPLETED	NO	1\2	50	2016/11/18-2023/12/31	PD-1	Nivolumab
NCT02829931	COMPLETED	NO	1	33	2016/8/22-2022/11/22	PD-1	Nivolumab
NCT04323046	RECRUITING	NO	1	20	2020/10/2-2029/3/1	PD-1	Nivolumab
NCT03991832	RECRUITING	NO	2	58	2019/12/31-2025/3/31	PD-L1	Durvalumab
NCT02794883	COMPLETED	YES	2	36	2016/11/1-2020/6/17	PD-L1	Durvalumab
NCT02336165	COMPLETED	YES	2	159	2015/2/26-2021/7/6	PD-L1	Durvalumab
NCT02866747	ACTIVE ;NOT_RECRUITING	NO	1\2	108	2017/1/17-2026/4/1	PD-L1	Durvalumab

### Combination therapy based on anti-PD-1/PD-L1

Increasing evidence suggests that combination therapies based on anti-PD-1/PD-L1 inhibitors can significantly improve the disease status and prognosis of glioma patients. Current combinations of PD-1/PD-L1 immunosuppressants in glioma treatment primarily involve several key aspects.

#### Combination therapy with chemotherapy

Cyclophosphamide is a chemotherapeutic agent that can induce lymphopenia and myelosuppression. The main mechanism of Cyclophosphamide is to induce DNA damage through methylation of DNA bases. It can react with purine bases in DNA by being converted into active methylidenes, thereby causing DNA strand breaks and base damage. This further leads to apoptosis and cell death [59]. In addition, the cytotoxic effect of Cyclophosphamide can also promote antigen presentation and enhance anti-tumor response [60]. When used in combination with PD-L1 inhibitors, Cyclophosphamide can induce apoptosis of tumor cells by triggering DNA damage. At the same time, PD-L1 inhibitors can activate the immune system and enhance the ability of T cells to attack tumor cells. Compared with PD-1/PD-L1 inhibitor monotherapy, the combination therapy of local Cyclophosphamide and PD-1 inhibitors resulted in longer survival and higher circulating lymphocyte count in patients. There is preclinical evidence indicating a cross-linking relationship between Cyclophosphamide treatment and PD-L1 downregulation at the GBM cell and tissue levels, and clinical studies are also ongoing on patients treated with Cyclophosphamide maintenance therapy and anti-PD-1 inhibitors in combination [61].

#### Combination with radiotherapy (RT)

The combination of PD-1/PD-L1 inhibitor and RT is also an option. RT can act on the TME of glioma and affect the growth and invasion of tumors. Both RT and PD-1/PD-L1 pathways act on glioma TME to influence tumor growth and invasion. RT can promote DC maturation and promote the recruitment of CD8^+^T cells into the tumor [62]. RT can also induce immunogenic death of tumor cells and reprogram TME by recruiting and activating effector T cells.Moreover, RT can weaken the inhibitory phenotype of Tregs. RT can inhibit the proliferation of Treg [63]. These effects are very similar to those produced by blocking the PD-1/PD-L1 pathway. Related study has shown that the combination of anti PD-1 immunotherapy and stereotactic radiosurgery (SRS) can increase cytotoxic T cell infiltration, reduce regulatory T cell levels, and prolong the median survival of mice compared to single therapy [55]. Therefore, the combination of anti PD-1/PD-L1 axis therapy and SRS may be a feasible treatment strategy for glioma.

#### Combination with oncolytic viruses (OV)

The combination of oncolytic viruses and PD-L1 inhibitors has emerged as a promising glioma treatment, offering new hope to patients. Research indicates that this strategy may enhance treatment efficacy and extend patient survival [64]. Oncolytic viruses can stimulate immune responses, effectively counteracting the immunosuppressive microenvironment of GBM, and synergizing with immune checkpoint inhibitors to regulate the glioma TME and activate anti-glioma immunity [65]. Several oncolytic viruses, including adenovirus, poliovirus, and retroviral vectors, are in various stages of clinical trials, showing promise in treating glioma patients [66]. Meanwhile, the application of PD-1 /PD-L1 immunosuppressants can alleviate the inhibitory effect of tumor cells on immune cells, enhance immune response, and make it easier for the immune system to clear tumor cells. The combination of oncolytic virus therapy and PD-1/PD-L1 inhibitors can achieve objective remission and even long-term survival in some recurrent GBM patients [67].

#### Chimeric antigen receptors (CAR)-T cell therapy

CAR-T cell therapy is a rising star in the tumor immunotherapy field. Briefly, T cells are genetically engineered with a specific CAR sequence resulting in recognizing tumor-specific antigens and killing tumor cells. At present, CAR-T cell therapy has achieved remarkable success in the treatment of hematological tumors, but there are still challenges in the treatment of solid tumor such as glioma [68]. At present, CAR-T cell therapies targeting different targets in glioma are undergoing related clinical trials, such as therapies targeting targets such as IL13Ra2 HER2 and EGFR(ref). These therapies have shown some efficacy in prolonging patient survival rate and period [69]. The use of PD-1/PD-L1 inhibitors in combination with CAR-T cell therapy aims to enhance therapeutic efficacy through a dual mechanism. Basically, PD-1/PD-L1 inhibitors relieve the functional inhibition of both T cells in TME, enhancing the ability of T cells to attack tumors. On the other hand, infused CAR-T cells are also de-suppressed by PD-1/PD-L1 inhibitors, thus increasing tumor attacking functionality. Meanwhile, PD-1/PD-L1 inhibitors change the tumor microenvironment and improve the immune response by releasing cytokines. And the combination of PD-1/PD-L1 immunosuppressants and CAR-T cell therapy has achieved significant progress in the clinical treatment of solid tumors [70]. This study provides an important reference and enlightenment for the combination of PD-1/PD-L1 immunosuppressants and CAR-T cell therapy in glioma, and supports the rationality of exploring similar combination therapy strategies in glioma.

The combination of PD-1/PD-L1 inhibitors and CAR-T cell therapy for the treatment of glioma holds immense promise for the future of cancer treatment. Future research will focus on exploring the precise identification of glioma-specific antigens, enhancing the survival and activity of CAR-T cells within the glioma microenvironment, and the potential for combining with other immunotherapeutic or targeted agents, aiming to overcome treatment challenges and improve patient prognosis.

#### Combination therapy of PD-1/PD-1 immunosuppressants and other immune checkpoint inhibitors

The regulation of TME in gliomas not only depends on various immune infiltrating nuclear immune cytokines, but also on the expression of various immune checkpoint receptors, such as Cytotoxic T lymphocyte associated antigen 4(CTLA-4) and PD-1/PD-L1 [71]. Related studies have shown that blocking a single checkpoint of PD-1/PD-L1 is insufficient to activate suppressed immune responses in gliomas [72], which has a higher degree of immunosuppression in TME. Therefore, it is urgent to explore the combination therapy of multiple immune checkpoint blocking. Table 2 Pathways and main application areas of immune checkpoint inhibitors.

**Table 2:** Pathways and main application areas of immune checkpoint inhibitors

Inhibitors	Mechanism of action	Immune checkpoint inhibitors	Site of action	Clinical status	Main application fields
PD-1/PD-L1 inhibitors	Block the binding of PD-1 and PD-L1, relieve the suppressive state of T cells, and enhance the anti-tumor immune response	Pembrolizumab	Acts on PD-1	approved for certain cancer types	Melanoma, non-small cell lung cancer, etc.
		Nivolumab	Acts on PD-1	approved for certain cancer types	Melanoma, non-small cell lung cancer, etc.
		Atezolizumab	Acts on PD-L1	approved for certain cancer types	Lung cancer, urothelial carcinoma, etc.
CTLA-4 inhibitors	Block the binding of CTLA-4 to CD80/CD86, relieve the suppressive state of T cells, and enhance the anti-tumor immune response	Ipilimumab	Acts on CTLA-4	approved for certain cancer types	Melanoma, renal cell carcinoma, non-small cell lung cancer
		Tremelimumab	Acts on CTLA-4	In clinical trials	Melanoma, liver cancer, non-small cell lung cancer
LAG-3 inhibitors	Block the binding of LAG-3 to MHC-II or other ligands, relieve the suppressive state of T cells, and enhance the anti-tumor immune response	Relatlimab	Acts on LAG-3	In clinical trials	Melanoma, non-small cell lung cancer, etc.
		MK-4280	Acts on LAG-3	In clinical trials	Multiple advanced solid tumors
		BMS-986,253	Acts on LAG-3	In clinical trials	Multiple advanced solid tumors
TIGIT inhibitors	Block the binding of TIGIT to CD155 or other ligands, relieve the suppressive state of T cells, and enhance the anti-tumor immune response	Antictumab	Acts on TIGIT	In clinical trials	Multiple cancer types
		Tiragolumab	Acts on TIGIT	In clinical trials	Melanoma, non-small cell lung cancer, etc.
Chemotherapy drugs	Interferes with the normal dynamics of microtubules and promotes tumor cell death	Paclitaxel		approved for certain cancer types	Breast cancer, ovarian cancer, etc.
	Interferes with the normal dynamics of microtubules and promotes tumor cell death	Docetaxel		approved for certain cancer types	Breast cancer, non-small cell lung cancer, etc.
	Interference DNA replication and repair	Cisplatin		approved for certain cancer types	Testicular cancer, ovarian cancer, lung cancer, etc.
	Interference DNA replication and repair	5-Fluorouraci		approved for certain cancer types	Colorectal cancer, gastric cancer, etc.
TIM-3 inhibitors	Blocking the binding of TIM-3 to Galectin-9 relieves the suppressive state of T cells and enhances the anti-tumor immune response	LY3415244	Acts on TIM-3 and PD-L1	In clinical trials	Advanced solid tumors
		Cobolimab	Acts on TIM-3	In clinical trials	Non-small cell lung cancer, etc.

##### CTLA-4

CTLA-4 is a protein receptor that acts as an immune checkpoint and downregulates the immune response. CTLA-4 can be induced to be highly expressed on the surface of activated CD4 + and CD8 + T cells, as well as CD4 + CD25 + FOXP3 + regulatory T cells (Tregs), which enhances immune suppression of T cells [73]. CTLA4 binds competitively with CD28 to CD80 and CD86, preventing CD28 from binding to these co-stimulatory molecules to activate T cells [74]. Study has shown that CTLA-4 inhibitors can restore T cell function and exhibit antitumor response when applied to mouse glioma models [75]. These results indicated that CTLA-4 inhibitors had a certain effect on the improvement of glioma. CTLA-4 and PD-1 are both receptors that Inhibitor of T cells to exert their anti-tumor effects [76]. PD-1/PD-L1 immunosuppressants and CTLA-4 immunosuppressants can induce T cell immune responses through different but complementary mechanisms. This process can promote the recovery of immune response in glioma TME (Fig. 2). In several cancers, combination therapy with immune checkpoint inhibitors (including anti-PD-1 and CTLA-4 antibodies) has shown higher therapeutic efficacy than single therapy [77, 78]. Compared with monotherapy in glioma, the combination of PD-1/PD-L1 immunosuppressants and CTLA-4 immunosuppressants can significantly prolong progression free survival in glioma patients [79].In the combined treatment of glioma, the systematic administration of CTLA-4, PD-L1, and PD-1 inhibitors can improve the survival rate of Patients with glioma [80].

##### Lymphocyte activation gene 3 (LAG-3)

LAG-3, as an immune checkpoint molecule, is primarily expressed on activated immune T cells within the tumor microenvironment [81]. LAG-3 can inhibit T cell activation by binding to MHC-II, as well as interacting with FGL-1, α-synuclein fibrils (α-syn), and the lectins galectin-3 (GAL-3) and lymph node sinusoidal endothelial cell C-type lectin (LSECtin), which reduces their ability to produce cytokines and proliferate, leading to the suppression of immune cell function (Fig. 2) [81]. This mechanism is analogous to the immunosuppressive pathway of PD-1/PD-L1. By co-blocking PD-1/PD-L1 and LAG-3, the inhibitory effects of these two immune checkpoints on T cells can be simultaneously lifted, thereby better restoring or enhancing T cell antitumor activity. Several studies have confirmed that LAG-3 inhibitors not only elicit a moderate therapeutic effect in cancer treatment but also enhance the efficacy of PD-1 inhibitors [82–84]. Related research has developed bispecific antibodies targeting both PD-L1 and LAG-3, which have demonstrated enhanced immunomodulatory capabilities in vitro and exhibited stronger antitumor activity. Although clinical trial data for the combined therapy of LAG-3 and PD-1/PD-L1 inhibitors in glioma are limited, the existing research suggests that this combination strategy shows potential in enhancing tumor-specific immunity [85].

##### T cell immunoreceptor with ig and ITIM domains(TIGIT)

TIGIT is a novel checkpoint inhibitory molecule that has emerged as an especially attractive target for cancer immunotherapy due to its significant role in constraining antitumor responses. Within the glioma tumor microenvironment (TME), TIGIT can inhibit the immune response through multiple mechanisms, including suppressing dendritic cell maturation through interaction with CD155, as well as enhancing the suppressive functions of Treg cells, thereby inhibiting the activity of various immune cells (Fig. 2) [86]. PD-1/PD-L1 and TIGIT were previously thought to suppress immune responses by regulating different costimulatory receptors (CD28 and CD226). However, study has shown that PD-1 and TIGIT employ distinct mechanisms that lead to the decreased or absent phosphorylation of CD226 on cytotoxic T lymphocytes (CTLs), resulting in a dysfunctional phenotype within the TME [87]. The co-administration of TIGIT and PD-1/PD-L1 inhibitors results in significantly lower phosphorylation of CD226 than when inhibitors are applied individually [88]. Therefore, only the combined application of TIGIT and PD-1/PD-L1 inhibitors can fully restore the normal expression of CD226, providing a mechanistic rationale for the combined treatment with PD-1/PD-L1 and TIGIT inhibitors. In tumor-associated immunotherapy research, the combination of anti-TIGIT and anti-PD-1/PD-L1 therapy has demonstrated good safety, enhanced antitumor activity, and improved survival benefits compared to anti-PD-1/PD-L1 treatment alone [89, 90]. For glioma, the use of combined anti-PD-1 and anti-TIGIT immunotherapy significantly improved survival rates in mouse glioma models compared to monotherapy [91]. Therefore, the combined treatment with TIGIT and PD-1/PD-L1 inhibitors holds great potential for enhancing the efficacy of immunotherapy for glioma and improving the prognosis of glioma patients.

##### T cell immunoglobulin and mucin domain-containing protein 3(TIM-3)

T cell immunoglobulin and mucin domain-containing protein 3(TIM-3) and its ligand galectin-9(GAL-9) are another option for immune checkpoint therapy. TIM-3 is a co-suppressor receptor that can be expressed on IFN-G-producing T cells, FoxP3^+^Treg cells, and innate immune cells (macrophages and dendritic cells) [92, 93],and its ligand GAL-9 is often highly expressed in glioma tissues [94]. TIM-3 has also been shown to suppress the immune response when interacting with its ligand GAL-9 [93]. At the same time, study has shown that in patients with malignant glioma, the expression of GAL-9 is higher in the tumor core than in the tumor periphery, and the survival of patients with high expression of GAL-9 is significantly shorter than that of patients with low expression [95]. This suggests that the TIM-3/GAL-9 pathway is closely related to the prognosis of glioma patients and plays a key role in the malignant progression of glioma [95, 96]. In several preclinical tumor models, the synergistic effect of immune checkpoint TIM-3 inhibitor, immune checkpoint PD-1/PD-L1 inhibitor, and CTLA-4 to block tumor cells in vivo to enhance anti-tumor immunity and inhibit tumor growth [93]. Moreover, the application of PD-1/PD-L1 immunosuppressants in glioma patients is accompanied by the development of drug resistance. Research has shown that resistance to PD-1/PD-L1 inhibitor monotherapy is associated with upregulation of immune checkpoint TIM-3 in lung adenocarcinoma [97]. Therefore, the combination therapy of PD-1/PD-L1 and TIM-3 immune checkpoints may be an effective treatment solution for patients who develop resistance to PD-1 /PD-L1 immunosuppressive therapy in the future. This combination therapy may be applied in immunotherapy for gliomas in the future. This suggests that TIM-3/GAL-9 immune checkpoints and PD-1/PD-L1 immune checkpoints have the potential to co-improve and treat glioma patients in the future.

At present, the main research direction of PD-1/PD-L1 axis in the field of gliomas is focused on the feasibility of combination therapy and related diagnostic and therapeutic methods.Whether it is combined with radiotherapy and chemotherapy, or other immunomodulatory therapies such as CTLA-4, or other treatment methods, the combination of action pathways will become one of the key focuses in the future diagnosis and treatment of glioma.

### Recent advances in predictive biomarkers in glioma

Biomarkers provide cancer-related information that is instrumental in predicting critical aspects of the disease, such as aiding in early detection and diagnosis, as well as assessing response to treatment and prognosis. This is crucial for improving clinical outcomes for patients and has long been a research objective for the development of biomarkers. Moreover, biomarkers can also contribute to a deeper understanding of tumor characteristics. There are also challenges in the spatial and temporal heterogeneity of PD-L1 expression, molecular subtype differences, and the combination with other biomarkers [98]. Therefore, future research needs to delve into the regulatory mechanisms, functional roles, and interactions with other immune-suppressive mechanisms of PD-L1 expression levels. Additionally, the development of more precise detection methods and more effective treatment strategies is essential to achieve precision and personalization in the immunotherapy of glioma. Although PD-L1 expression levels are associated with the therapeutic response to PD-1/PD-L1 inhibitors in some cancers [99], it is not an absolute predictor. In gliomas, PD-L1 expression levels may be an important predictor, but other biomarkers and specific patient characteristics also need to be considered.

Tumor Mutation Burden (TMB) acts as a biomarker for the sensitivity to immune checkpoint inhibitor responses, with high TMB generating a greater number of tumor neoantigens recognizable by T cells. When PD-1 checkpoint is inhibited, it may result in a more potent anti-tumor immune response. TMB has been approved by the FDA as a companion diagnostic biomarker for the PD-1 inhibitor pembrolizumab [100]. Study has indicated that in various cancers undergoing immunotherapy, patients with higher TMB can achieve a more favorable prognosis [101]. In the context of PD-1/PD-L1 immunotherapy for glioma, TMB also holds significant research value as a biomarker. Research has shown that TMB is inversely correlated with overall survival in glioma patients, and high TMB may suppress immune infiltration, potentially leading to increased glioma infiltration and growth [102]. TMB has emerged as a biomarker with potential value for glioma. As research in this area deepens, studies have found that TMB may assist clinicians in identifying patients who could benefit from immunotherapy and could pave the way for the development of more valuable personalized immunotherapy treatment plans for glioma patients in the future [103, 104].

There are still many predictive biomarkers in glioma that are being studied, such as microRNA, microsatellite instability, etc. The research of these predictive biomarkers will continue to deepen, which is expected to provide more efficient and accurate diagnosis and treatment methods for clinical practice, and bring better quality of life and prognosis to glioma patients.

### The blood-brain barrier (BBB)

BBB is a protective barrier that shields the brain from potentially harmful substances in the blood, but the PD-1/PD-L1 immune checkpoint blockade therapy is also restricted by the BBB in the treatment of gliomas. PD-1 and PD-L1 antibodies are relatively large molecules that may not achieve high intratumoral concentrations due to the BBB, which could reduce their therapeutic benefits. To overcome this barrier, researchers have developed ferritin-based nanocages that can effectively cross the BBB and target brain tumors, significantly inhibiting PD-1/PD-L1 interaction and restoring T-cell activity [105]. Additionally, a research team has developed a new formulation of PD-L1 monoclonal antibody, MB-aPDL1. This formulation is capable of crossing the BBB and precisely releasing the antibody to the tumor microenvironment, thereby improving the tumor immune microenvironment and enhancing therapeutic efficacy [106]. These studies indicate that although the BBB affects to PD-1/PD-L1 immunotherapy, these challenges are being gradually overcome through innovative drug delivery systems and treatment strategies.

## Challenges and potentials

The role of the PD-1/PD-L1 axis in gliomas is very complex. It involves not only the interaction between PD-1 and PD-L1, but also with a variety of immunosuppressive molecules and cells that work together to shape the immunosuppressive microenvironment of glioma, for example, the polarization state of GAMs, the functional status of TILs, and the number and activity of Tregs all affect the function of the PD-1/PD-L1 axis. These studies will contribute to the development of more effective targeted therapies for glioma. Given the limited efficacy of PD-1/PD-L1 monotherapy in glioma, further development and optimization of combination therapy strategies will be carried out in the future, including exploring the most effective combination, dose and timing of treatment. Combination therapy can overcome the limitations of single therapy and improve treatment outcomes. Combination therapy strategies include chemotherapy, radiotherapy, oncolytic virus, CAR-T cell therapy, and combinations with other immune checkpoint inhibitors, which are showing promise.

The study of PD-1/PD-L1 inhibitors in the treatment of glioma must overcome numerous challenges, because glioma is a highly heterogeneous tumor, with PD-1/PD-L1 expression levels, immune cell composition, and response to treatment varying among different patients. Therefore, a personalized treatment plan tailored to each patient’s specific condition is essential to improve treatment outcome. The immunosuppressive microenvironment of glioma is one of the major challenges for PD-1/PD-L1 inhibitors in the treatment of glioma patients. This environment contains a variety of immunosuppressive cells and molecules that work together to inhibit anti-tumor immune responses, such as regulatory T cells (Tregs), myeloid-derived suppressor cells (MDSCs), and tumor-associated macrophages (GAMs) [26, 35, 42]. In addition, the blood-brain barrier is currently one of the leading causes affecting PD-1/PD-L1 inhibitor therapy in glioma patients. In summary, these challenges are crucial for improving treatment outcomes and the quality of life for patients, and they also represent the future research direction for PD-1 and PD-L1.

## Data Availability

Not applicable.
